# Spina Bifida: Pathogenesis, Mechanisms, and Genes in Mice and Humans

**DOI:** 10.1155/2017/5364827

**Published:** 2017-02-13

**Authors:** Siti W. Mohd-Zin, Ahmed I. Marwan, Mohamad K. Abou Chaar, Azlina Ahmad-Annuar, Noraishah M. Abdul-Aziz

**Affiliations:** ^1^Department of Parasitology, Faculty of Medicine, University of Malaya, 50603 Kuala Lumpur, Malaysia; ^2^Laboratory for Fetal and Regenerative Biology, Colorado Fetal Care Center, Division of Pediatric Surgery, Children's Hospital Colorado, University of Colorado, Anschutz Medical Campus, 12700 E 17th Ave, Aurora, CO 80045, USA; ^3^Training and Technical Division, Islamic Hospital, Abdali, Amman 2414, Jordan; ^4^Department of Biomedical Science, Faculty of Medicine, University of Malaya, 50603 Kuala Lumpur, Malaysia

## Abstract

Spina bifida is among the phenotypes of the larger condition known as neural tube defects (NTDs). It is the most common central nervous system malformation compatible with life and the second leading cause of birth defects after congenital heart defects. In this review paper, we define spina bifida and discuss the phenotypes seen in humans as described by both surgeons and embryologists in order to compare and ultimately contrast it to the leading animal model, the mouse. Our understanding of spina bifida is currently limited to the observations we make in mouse models, which reflect complete or targeted knockouts of genes, which perturb the whole gene(s) without taking into account the issue of haploinsufficiency, which is most prominent in the human spina bifida condition. We thus conclude that the need to study spina bifida in all its forms, both aperta and occulta, is more indicative of the spina bifida in surviving humans and that the measure of deterioration arising from caudal neural tube defects, more commonly known as spina bifida, must be determined by the level of the lesion both in mouse and in man.

## 1. Introduction

Spina bifida is the most common and complex central nervous system malformation in humans. Management of these patients involves various disciplines to ensure the best possible outcome achieved and provide a good quality of life for its patients [1, 2]. The study of this condition is extremely relevant in that even in the 20 years since the discovery of the benefits of folic acid this condition is highly prevalent around the world and its occurrence does not seem to decrease [3]. Interestingly, the debate is very much ongoing upon the evidence that the United States of America has seen a decline in cases of spina bifida (https://www.cdc.gov/ncbddd/spinabifidadata.html). This review paper intends to compare and contrast spina bifida in humans and spina bifida in the mouse, which is the leading animal model of this devastating condition in light of the information studies on animal models have shed on the human counterpart [4–6].

## 2. Spina Bifida in Humans

Development of the central nervous system including the brain and spinal cord is a complex process beginning with a flat sheet of cells which undergoes sequential thickening, elevation, mediolateral convergence accompanied by rostrocaudal extension, and finally adhesion to form the neural tube (NT) which is the precursor of the brain and the spinal cord. Perturbations of these interconnected processes result in neural tube defects (NTDs), which are the most common congenital malformation affecting this system and are associated with significant complications. NTDs can occur in two major forms: spina bifida (SB) aperta, which is the open-lesion NTD, and the closed-lesion NTD, more commonly known as SB occulta.

## 3. Epidemiology

Spina bifida is the most common nonlethal malformation in the spectrum of NTDs and has an incidence generally around 0.5 per 1,000 births, although higher frequencies have been reported [7–11]. In the United Kingdom, the population prevalence of spina bifida is 7.8–8.4 per 10,000 for males and 9.0–9.4 per 10,000 for females [12]. While the prevalence in the United States of America is more than 3 in every 10,000 births [8, 13], studies in parts of Asia, such as Malaysia, have also shown a lower occurrence of spina bifida than that of the UK [14]. More recent efforts by our group (“Spina Bifida: A 10-Year Retrospective Study at University of Malaya Medical Centre, Malaysia,” manuscript in submission), however, have found that the lower rate of NTDs may not be completely representative as in our hospital alone from the years 2003 to 2012 we have had over 10 cases of neural tube defects per year (spina bifida and anencephaly). Furthermore, certain regions of China have shown much higher preponderance of this condition than in other parts of the world [15–18]. In Africa, for example, spina bifida has been recorded as being low in occurrence in comparison to other birth defects but questions have arisen with regard to record-taking and data management [19]. Gender preponderance differs according to country; in the USA, spina bifida is thought to be more prevalent in girls than in boys [20, 21].

## 4. Pathogenesis

**Figure 1:**
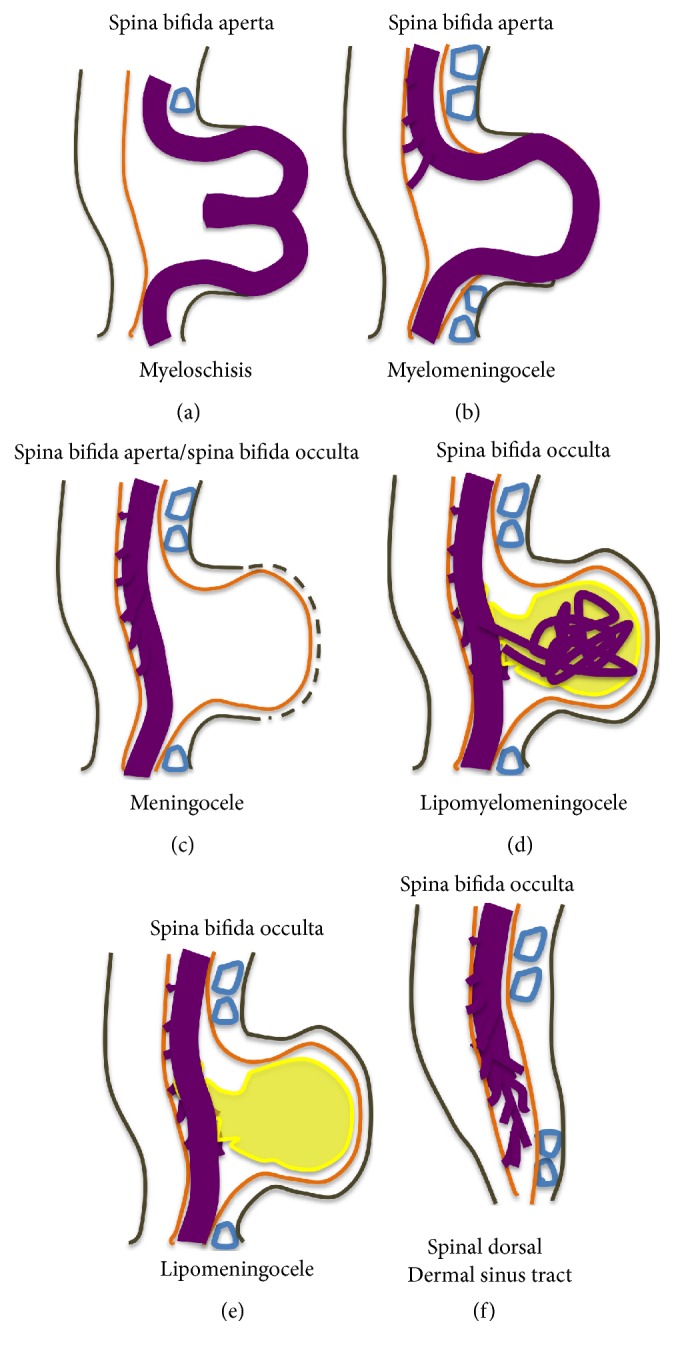
Schematic representation of the open (aperta) and close (occulta) types of spina bifida. (a) Myeloschisis which represents the most severe form of open spina bifida. (b) Myelomeningocele which represents another typical severe form of open spina bifida (spina bifida aperta/spina bifida cystica). The typical representation is that of the spinal cord lying outside the spinal canal. (c) Meningocele that represents open or close spina bifida (the skin may or may not be present) but spinal cord does not lie outside the spinal canal. (d) Lipomyelomeningocele that represents closed spina bifida (spina bifida occulta) (covered with skin) but spinal cord is intermeshed with lipid globules (in yellow). (e) Lipomeningocele that exhibits closed spina bifida but spinal cord does not lie outside spinal canal even though lipid globules are present. (f) Spinal dorsal dermal sinus tract; spina bifida occulta with vertebral arches missing (often asymptomatic and is thought to be a mesodermal defect and a defect of secondary neurulation).

*Spina bifida aperta (SBA)*, sometimes referred to as spina bifida cystica, is usually visible at birth as an exposed neural tissue with or without a protruding sac at the site of the lesion. SBA may be referred to as either myeloschisis (Figure 1(a)) or myelomeningocele (Figure 1(b)). Myelomeningocele is when the spinal cord protrudes from the spinal canal into a fluid-filled sac resulting from incomplete closure of the primary neural tube. Myeloschisis is when the incomplete closure of the primary neural plate results in a cleft spinal cord with the edges flush with the defect. The extent and severity of the neurological deficits depend on the location of the lesion along the neuraxis [22].

Meningocele (Figure 1(c)) is often described as a less severe variant of myelomeningocele in which the spinal cord is not found in the sac and is described by embryologists to be absent of neural matter in its herniated sac; and its description is often coupled with that of myelomeningocele which clearly has neural matter herniating at the site of the open lesion. Therefore, the status of meningocele being an open (aperta) or closed (occulta) defect is still debatable in terms of embryogenesis. However, imaging evidence by radiologists has firmly placed meningocele as spina bifida occulta [3, 7, 121–123].

Myelomeningocele (MMC) is usually associated with a type II Chiari hindbrain malformation, ventriculomegaly, and hydrocephalus [124, 125]. Chiari type II malformation is the downward displacement of the cerebellar vermis into the cervical vertebral canal [22, 125]. It is often symptomatic and is diagnosed prenatally with ultrafast fetal magnetic resonance imaging (MRI) [126, 127]. This malformation causes elongation of the brain stem and obliteration of the fourth ventricle, leading to obstruction of cerebrospinal fluid circulation and development of hydrocephalus in 90% of patients [22]. Treatment of such accompanying hydrocephalus is needed in about 82% of cases and involves draining of cerebrospinal fluid into either the peritoneal or other body cavity via a subcutaneous shunt [128].

*Spina bifida occulta (SBO)* is the second major form of NTDs, where the site of the lesion is not left exposed [129, 130]. Spina bifida occulta encompasses lipomyelomeningocele (Figure 1(d)), lipomeningocele (Figure 1(e)), and spinal dorsal dermal sinus tract (Figure 1(f)) ranging phenotypically from (i) dysplastic skin, (ii) tuft of hair, and (iii) vestigial tail as well as other forms of spinal dysraphism, which lack a pathogenic representation when the vertebrae develop abnormally leading to absence of the neural arches [131, 132]. In symptomatic cases, tethering of the spinal cord within the vertebral canal can result in pain, weakness, and incontinence in otherwise normal, healthy children or adults [133].

## 5. Treatment and Management

Management of patients with myelomeningocele has improved drastically from the mid-1970s when patients were sometimes denied treatment based on the severity of their condition [134] to the current state-of-the-art prenatal in utero repairs performed at highly specialized centers [127, 128]. Neonatal surgical closure of the lesion is considered the standard of care against which all novel management options are compared [22, 135, 136].

NTDs have a profound impact on society. The morbidity and mortality rates of spina bifida patients decrease with improving medical care. Taking the United Kingdom as an example, Bowman et al. [137] in their 25-year follow-up of 71 spina bifida aperta patients found that at least 75% of these children can be expected to reach their early adult years [137]. Moreover, as many as 85% are attending or have graduated from high school and/or college. More than 80% of young adults with spina bifida have social bladder continence. In the same study, 49% had scoliosis, with 43% eventually requiring a spinal fusion. Approximately one-third of patients were allergic to latex, with six patients having experienced a life-threatening reaction. Renal failure was 6.8–9.0 times more common for males and 9.2–11.5 times more common for female patients compared with the general population in each of the years 1994–1997 in the UK [138]. Therefore, longer life equates with the need for progressively better quality of life.

The sequelae of NTDs are staggering and appear to have not only anatomical effects secondary to the primary defect but also functional, emotional, and psychological morbidities including bladder and bowel incontinence, paralysis, musculoskeletal deformity, and shunt malfunctions and infections, among others. Moreover, the costs involved in maintenance of spina bifida patients include mobility aids (orthoses, wheelchairs, and crutches), medications, and the cost associated with shunt revisions, in addition to the cost of modifications to public utilities that are required to enable disabled access. Ultimately, its compound nature results in an immense financial burden amounting to $1,400,000 per child affected by NTD over a 20-year life span [139–142].

### 5.1. Syndromic and Nonsyndromic (Isolated) Spina Bifida

A small proportion of NTDs in live born infants are associated with specific syndromes that are associated with chromosomal or single-gene disorders [143]. NTDs are currently considered as “complex” disorders with genetic and environmental factors playing roles in causation [144], which have been summarized in Table 1. Craniorachischisis and encephalocoele have the highest rate of syndromic association, anencephaly and high spina bifida have intermediate rates, and caudal spina bifida has the lowest rate [145]. The role of folic acid in preventing syndromic NTDs turned out to be not as gratifying as for nonsyndromic (isolated), multifactorial NTDs [146]. It should be noted that while syndromic NTDs may have identifiable genetic causes, many of the nonsyndromic (isolated) NTDs have unidentified genetic etiology. Most of human neural tube defects are nonsyndromic with NTD being the only defect. The focus of this review paper is on nonsyndromic (isolated) spina bifida apart from the clearly stated syndromic spina bifida mentioned specifically in Table 1.

### 5.2. Causative Factors, Detection, and Prevention of Spina Bifida

The etiology of spina bifida is heterogeneous [147–150]. Most nonsyndromic spina bifida is thought to be of multifactorial origin [151] with influence of both genetic and environmental factors [144, 152]. Among the environmental factors associated with increased risk of spina bifida are increased pregnancy weight [153–158], maternal smoking [159–161], drug intake specifically of antiepileptic drugs [162–164], and maternal illnesses such as diabetes [165, 166] and hyperthermia [167]. Dietary factors including water chlorination [168–170], inositol intake [171], simple sugar intake [172], and the intake of trace elements and other micronutrients [173–176] have been proposed to act as either contributory or preventive factors for spina bifida. Isolated spina bifida is caused by cytogenetic abnormalities in 2–16% of cases [177–179].

Elevated levels of maternal serum alpha-fetoprotein are usually indicative of spina bifida aperta [180, 181] but can be associated with other conditions (e.g., twin gestation and abnormalities of placentation including placental lakes and placenta previa) and ultrasound is needed to confirm the diagnosis. Screening obstetrical ultrasonography is the initial routine method for the detection of NTDs during pregnancy in many countries. However, it sometimes fails to detect closed spina bifida [182, 183]. In highly specialized fetal centers, use of ultrafast fetal MRI has enabled detailed anatomical evaluation of the defect and accurate assessment of its accompanying effects [126].

It has been over 15 years since the Medical Research Council Vitamin Trial involving 33 centers around the world conclusively showed that 72% of recurrent NTD cases could be prevented by folic acid supplements in the periconceptional period [184]. A further study [185] showed that the first occurrence of spina bifida could also be prevented by folic acid. However, not all NTDs are responsive to folic acid and inositol has been shown as a possible additional therapy, based on prevention of spina bifida in folate-resistant NTDs in mice as well as the PONTI human trial [186, 187]. Calcium formate too has been shown to have preventive effects on NTD in mice but evidence is not yet forthcoming in prevention of human NTDs [188–190]. There still remains room to study whether there are other supplements out there that can prevent spina bifida.

## 6. Surgical Management of Spina Bifida

Surgical management of spina bifida here is discussed as a 2-point discussion: first is surgical management prior to the advent of in utero repair of open spina bifida and second is in utero repair leading to the Management of Myelomeningocele Study (MOMS) trial [128]. Postnatal repair of open spina bifida repair is a requirement in order to prevent further mechanical damage and infection. The lesion either may be closed primarily with the aid of skin and muscle flaps or may require a synthetic patch such as AlloDerm (LifeCell Corp., Branchburg, NJ) [191], gelatin, or collagen hybrid sponges [192]. In utero MMC repair in humans was first reported in the landmark paper published in 1998 [127]. However, since then, a handful of centers have been offering in utero repair. Furthermore, its popularity has increased in Europe [193]. The principle of in utero repair is to prevent the 2-hit hypothesis much described in previous literature that the child is exposed to neurological deterioration contributed first by failure of the neural tube to form and secondly by physical and chemical perturbation inflicted on the exposed neurological tissue of the open lesion [128, 194]. In an elegant experimental study, Meuli et al. [195] concluded that surgical exposure of the normal spinal cord to the amniotic space in a 75-day sheep fetus results in a MMC-type pathology at birth with clinical, histological, and morphological attributes comparable to human MMC. Heffez et al. [196] has demonstrated that spinal cord injury caused by exposure to the intrauterine milieu can be prevented by primary closure of the fetal skin incision as late as hours after creating the defect. It also demonstrated that ongoing exposure beyond 24 hours leads to spinal cord damage and permanent neurological deficit. Moreover, animal studies have previously shown that prenatal coverage of a spina bifida-like lesion preserves neurologic function and improves hindbrain herniation [195, 197, 198].

The first human prenatal repair of MMC was reported in Tulipan et al. [199]. Cumulative data suggested not only a dramatic improvement in hindbrain herniation but also increased maternal and neonatal risks including preterm labor, uterine dehiscence, and increased risk of fetal and neonatal death among others. Adzick et al. [128] investigated the effects of prenatal repair of MMC via a randomized prospective study. It reported that prenatal surgery for MMC performed before 26 weeks of gestation decreased the risk of death or need for shunting by the age of 12 months and also improved scores on a composite measure of mental and motor function, with adjustment for lesion level, at 30 months of age. Prenatal surgery also improves the degree of hindbrain herniation associated with Chiari II malformation, motor function, and the likelihood of being able to walk independently, as compared with postnatal surgery [128]. Open prenatal repair comes with an increased maternal and neonatal risk including preterm labor, uterine dehiscence, premature rupture of membranes, and increased risk of fetal and neonatal death. The main goal for prenatal repair of MMC is to achieve skin closure to prevent further damage of the placode and arrest the CSF leak.

## 7. Human Spina Bifida Genes

Despite the 250 mouse mutants with NTDs to date, there has yet to be a significant breakthrough for human NTD gene(s) both causal and/or associated with NTDs that can be used for genetic screening worldwide [4, 7]. The importance of finding candidate gene(s) as a genetic screening tool for potential parents cannot be undervalued as it has been estimated that the total lifetime costs for patients with spina bifida (spinal NTDs) amount to about $1.4 million in the US and more than €500k in Europe, with 37.1% of the total cost attributed to direct medical costs and the remainder in indirect costs, including the needs of the caregiver [200].

Despite observation of multiplex nonsyndromic NTD cases in multigenerational NTD families as seen in 17 US and 14 Dutch families with more than 1 NTD-affected person, there are other NTD cases that are simplex and sporadic as seen in identical twins with lumbosacral lipomyelomeningocele with no known familiar history of NTDs [201, 202]. This suggests that NTDs have a multifactorial genetic etiology.

To date, the strongest candidate thus far for a potential NTD screening gene is the methylenetetrahydrofolate reductase* (MTHFR)* C677T (rs1801133) polymorphism in populations of non-Latin origin (meta-analysis study) [203]. In recent meta-analysis study, Zhang et al. support the significant association between C677T and NTDs in case-control studies (22 studies, 2,602 cases, and 4,070 controls) [204]. The second most studied* MTHFR* variant is A1298C, which did not report any significant increase in risk of NTDs [204]. Another meta-analysis study by Blom et al. (2006) reported increased risk in mothers and associated with NTD infants who are homozygous for C677T variant [205]. In spina bifida case studies,* MTHFR* C677T variant was clearly reported as associated gene or risk factors in Irish* (451 spina bifida patients)*, mixed USA, mixed UK, and Italian cohort but not in other 180 Dutch patients (Table 3), while A1298C variant was reported with no association to spina bifida cases in Italian, Mexican (Yucatan), and Dutch population (Table 3).* MTHFR* is the most studied human spina bifida gene, as its role in folate one-carbon metabolism fits into a clear mechanism of NTD. However, the studies have not been well replicated in many other populations across the world, indicating that it is not likely to be either a major contributor or a common factor in NTD globally.

Other genes such as the planar cell polarity (PCP) genes, which have been studied in spina bifida cohorts among Italians, Americans, and the French, are* VANGL1* and* CESLR1* [44, 82–84]. The noncore PCP gene* SCRIB* has also been implicated as a spina bifida gene among the American cohort [85]. However, noncore PCP gene association needs to be explored further in larger NTD cohorts. To date, over 100 human spina bifida genes have been used to screen for spina bifida with 48 genes reported as a potential risk factor as listed in Table 3 which was reviewed in Greene et al. [93]; further candidates since then are* NKX2-8, PTCH1, Glypican-5, PARD3, Paraoxonase 1, COMT, AMT*, and* GLDC* genes [16, 17, 206–211]. All of these do not represent a potential global spina bifida gene. Therefore, a strong candidate spina bifida gene(s) for the world population has yet to be discovered.

## 8. Spina Bifida in Mouse

There exist more than 250 mouse models with neural tube defects, of which 74 are of spina bifida (Table 2) [4], yet there does not exist a single mouse gene which can be used to screen the orthologous human gene of neural tube defect nor spina bifida to date [212]. That said, it does not mean that the studies on the structural changes afforded by the mouse model cannot be used as a tool to understand human spina bifida. We discuss the various studies on mouse neurulation below and why it is still an invaluable tool for understanding human neurulation.

### 8.1. Mechanisms of Neural Tube Closure

In vertebrates, the development of the CNS starts with the formation of the neural plate on the dorsal surface of the embryo during late gastrulation [213, 214]. A complex morphogenetic process transforms the neural plate into the hollow neural tube in a process known as* “neurulation”* [213]. Primary neurulation is responsible for formation of the neural tube throughout the brain and the spinal cord rostral to the mid-sacral level [215]. At more caudal levels, an alternative mechanism (secondary neurulation) operates whereby the neural tube is formed by canalization of a condensed rod of mesenchymal cells in the tail bud [216].

**Figure 2:**
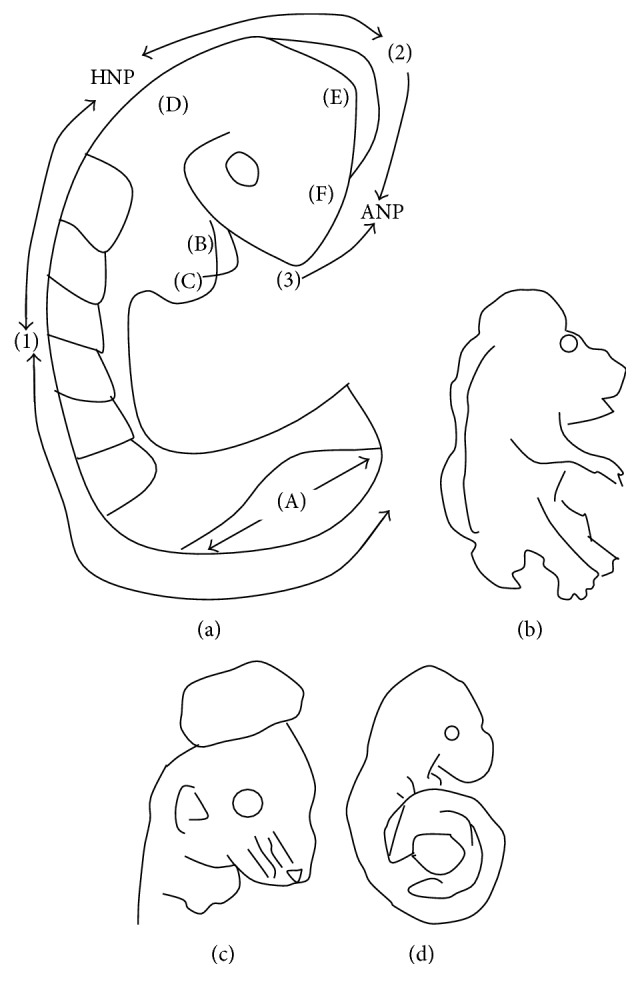
Points of closure in the mouse embryo and phenotypes of failure of closure of the various points along the neuraxis. (a) Schematic figure illustrating the multiple points of closure of the neural tube, directions of closure, and the different locations of neuropores in the developing embryo. (1), site of closure (1) which occurs at the level of somite 3 in the 6-7-somite embryo. Closure (1) is the initiation event of neurulation. Closure then progresses caudally and is completed by closure of the posterior neuropore (PNP) at the 29-30-somite stage of development; (2), second closure site at around the 10-somite stage; (3), closure (3) site which begins soon after closure (2). Arrows depict spreading of neural tube closure to neighbouring regions with completion of anterior neuropore closure soon after initiation of closure (3) and closure of the hindbrain neuropore at the 18–20-somite stage. (b) Phenotype resulting from failure of closure (1): craniorachischisis; (c) phenotype resulting from failure of closure (2): exencephaly; (d) phenotype of failure of the caudal wave of spinal closure, leading to an enlarged PNP and later development of spina bifida. (A), posterior neuropore; (B), branchial arches; (C), developing heart; (D), hindbrain; (E), midbrain; (F), forebrain; ANP: anterior neuropore; HNP: hindbrain neuropore.

The process of neurulation in mammals and some other vertebrates is considered discontinuous because it occurs simultaneously at multiple sites along the neuraxis [215–219]. There are three points of* de novo* neural tube fusion in the mouse, which is the most studied mammalian model ([220]; see Figure 2(a)).* Closure 1* occurs adjacent to somite 3 in embryos with 6-7 somites and progresses rostrally and caudally,* closure 2* occurs at the midbrain-forebrain boundary at around the 10-somite stage and progresses caudally, and* closure 3* occurs at the rostral end of the forebrain, soon after closure 2.

Considering this discontinuous process of neurulation, it can be understood why NTDs are such a complex group of heterogeneous birth defects, with various phenotypic presentations. Failure of* closure 1* leads to craniorachischisis (Figure 2(b)); failure of closures 2 and/or 3 causes exencephaly and/or anencephaly, respectively (Figure 2(c)), while failure of neurulation to progress from the site of closure 1 caudally along the spinal axis leads to spina bifida aperta (Figure 2(d)).

During neurulation, the neuroepithelium must undergo various structural changes in order to achieve closure. The advent of molecular biology has allowed scientists to identify the genes that are required for these structural changes to occur. The next section gives a brief overview of the research to date on how gene expression affects structural changes in neural tube development, with an emphasis on gene regulation in the spinal region.

### 8.2. The Structural Changes of the Mouse Neural Tube during the Process of Closure

**Figure 3:**
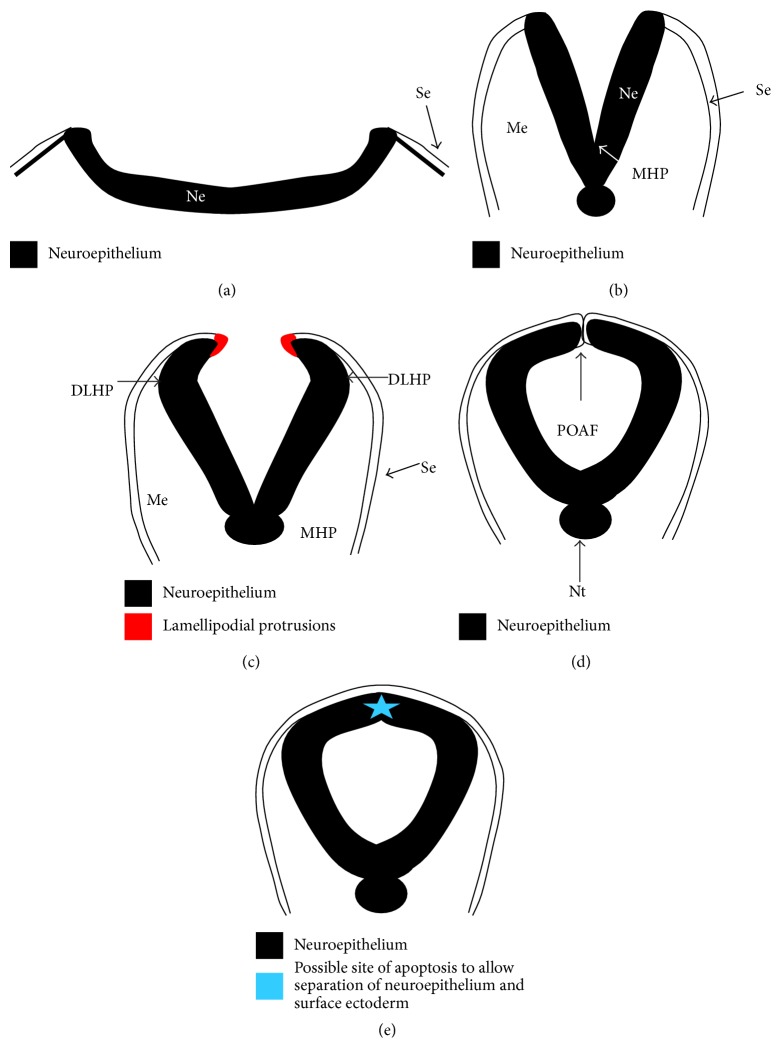
Schematic representation of the formation of the mouse spinal neural tube. Process of closure of the PNP of embryos undergoing Mode 1 (a, b, d) or Mode 2 (a, c, d) neurulation. (a) Neuroepithelium thickens and converges; (b) formation of bilateral neural folds which are elevated (Mode 1); (c) apposing tips of neural folds aided by bending at the dorsolateral hinge points (DLHP) of the bilateral neural folds (Mode 2); (d) adhesion and fusion at the tips of the neural folds; (e) remodeling of the neural tube. Ne, neuroepithelium; Se, surface ectoderm; Me, mesoderm; MHP, median hinge point; DLHP, dorsolateral hinge points; POAF, point of adhesion and fusion; Nt, notochord.

Morphologically, the mouse neural tube undergoes distinct structural changes prior to its closure [7, 215, 221–224]. A summary of the spatiotemporal expression of genes in the mouse neural tube during neurulation is as shown in Table 5. The neuroepithelium narrows and lengthens, a process referred to as convergent extension (Figure 3(a)), in which the polarized cells which form the neuroepithelial plate converge towards the midline, elongate anteroposteriorly, and then intercalate [215, 225].

**Figure 4:**
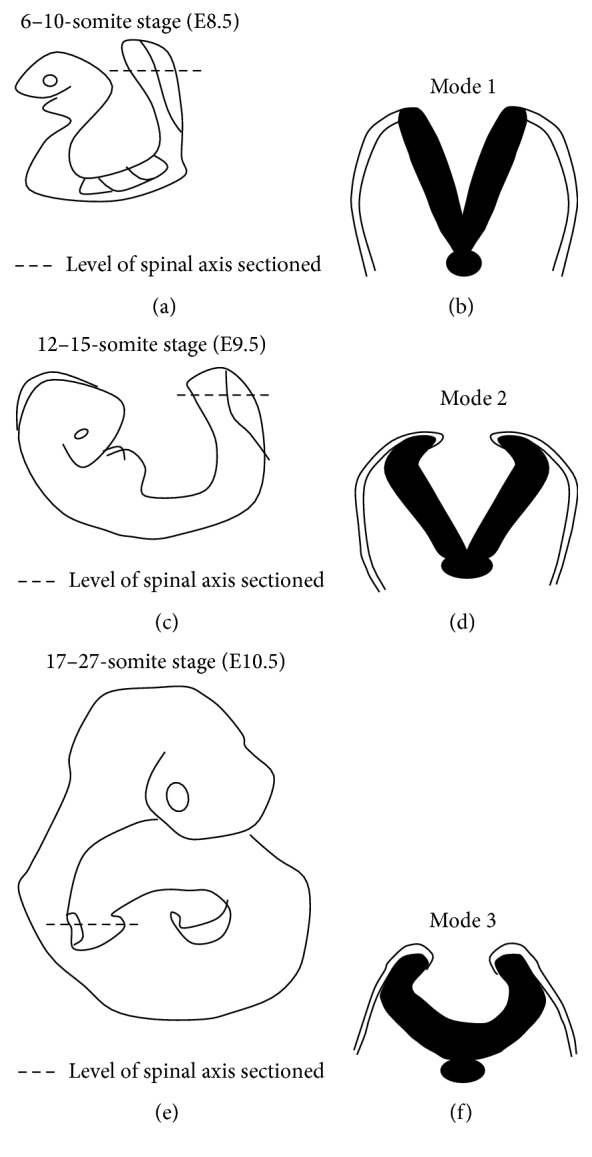
Schematic figure showing progressive developmental stages of the mouse embryo and sections through the PNP at these stages. (a) Schematic of embryo at 6–10-somite stage, which has already undergone closure (1); (b) section through PNP of (a), depicting Mode 1 neurulation; (c) schematic of embryo at 12–15-somite stage; (d) section through PNP of (c) exhibiting Mode 2 neurulation; (e) schematic of embryo which has undergone closures (1), (2), and (3) with PNP being the only remaining unfused section of the neural tube; (f) section through PNP of (e) depicting Mode 3 neurulation.

Convergent extension leads to narrowing and lengthening of the neuroepithelium, a process that has been suggested also to assist neural fold elevation via axial elongation [105, 226–228]. However, the lengthening of the body axis is disrupted by manipulation of gene function required for convergent extension; whilst the neural folds are still able to elevate, convergent extension still fails [227, 229, 230]. Hence, convergent extension and neural fold elevation are separable processes. Elevation of the neural folds at high levels of the spinal neuraxis results from the formation of a median hinge point (MHP) (Figure 3(b)) in a process termed Mode 1 neurulation [215, 231, 232]. The neural folds remain straight along both apical and basal surfaces, resulting in a neural tube with a slit-shaped lumen. Mode 1 neurulation occurs during formation of the spinal neural tube in 6–10-somite stage embryos, as shown in Figures 4(a) and 4(b).

A second set of hinge points are formed dorsolaterally at more caudal levels of the spinal neuraxis, the dorsolateral hinge points (DLHPs), a process that appears to enhance the ability of the apposing tips of the neural folds to come close to each other (Figure 3(c)). Mode 2 occurs during formulation of the spinal neural tube in 12–15-somite stage embryos and generates a diamond-shaped lumen, as depicted in Figures 4(c) and 4(d). In Mode 2, a median hinge point is also present, whereas the remaining portions of the neuroepithelium do not bend. At the 17–27-somite stage, the neural tube closes without a median hinge point, whereas dorsolateral hinge points are retained. This is known as Mode 3 neurulation and generates an almost circular shaped lumen, as shown in Figures 4(e) and 4(f).

Adhesion of the tips of the apposing neural folds is the final step in primary neurulation, enabling the neural tube to complete its closure [215]. The tips of the apposing neural folds and the eventual point of adhesion are reported to contain cell to cell recognition molecules (as demonstrated in red in Figure 3(c)) which may be required for the specific adhesion process to occur [233–243]. This is supported by previous evidence that the cell surface of the neuroepithelium is lined by carbohydrate-rich material that is not observed in the rest of the neuroepithelium [238]. Removal of GPI-anchored proteins from the cell surface during neurulation results in delayed spinal neural tube closure [244]. Interestingly, work performed by Abdul-Aziz et al. and Pyrgaki et al. demonstrated protrusions emanating from the neural fold tips that interdigitate leading to eventual adhesion [244, 245] (Figure 3(d)). Ultimately, the newly formed neural tube undergoes remodelling via apoptosis to enable the neural tube to separate from its surface ectoderm [228, 246] (Figure 3(e)).

### 8.3. Primary Neurulation Versus Secondary Neurulation

Primary neurulation and secondary neurulation are important developmental processes and have been described in many models. In the chick, there does not exist a clear distinction as to when primary neurulation ends and secondary neurulation begins; the lower spinal cord has been described as junctional neurulation, whereby ingression and accretion accompany the process of defining the area which straddles primary and secondary neurulation and is therefore thought to somehow represent human thoracolumbar spina bifida [247].

In mouse and humans, spina bifida occulta has largely been described as a result of failure of secondary neurulation [3, 215]. However, much has been described of the severity of lipomyelomeningocele [131, 248] in comparison to the somewhat neurologically unperturbed tethered cord phenomenon which is brought on by trapped nerves due to missing vertebral arches [133]. What is evident is that, irrespective of whether or not there is skin covering the neural tube defect lesion, the severity of the condition depends on the level where the site of the lesion is located. Secondary neurulation in the mouse is described as occurring at sacral level 2 [224]. Therefore, to describe lipomyelomeningocele as resulting from failure of secondary neurulation would be artificial.

## 9. The Genetics behind the Structural Changes in Spinal Neural Tube Closure

This section summarizes the various genes that are switched on during neurulation and whose functions have been implicated in the various structural changes that the spinal neural tube undergoes in order for closure to be achieved.

### 9.1. Planar Cell Polarity and Convergent Extension

Planar cell polarity (PCP) is a process in which cells develop with uniform orientation within the plane of an epithelium [249]. The PCP pathway is a noncanonical Wnt pathway [225, 250–252]. Various Wnt molecules are known to play roles in the PCP pathway such as Wnt11 and Wnt5a [250, 253].

PCP signaling has been suggested to be primarily required for cytoskeletal activity, for example, cellular protrusion, cell-cell adhesion, and cell-matrix adhesion [254]. Skin development, body hair orientation, polarization of the sensory epithelium in the inner ear, and the directed movement of mesenchymal cell populations during gastrulation are among the processes requiring proper PCP signaling in vertebrates [227, 254–256]. In vertebrates, function of the PCP pathway appears to be required for convergent extension (CE). Lamellipodia have been the type of cell shown to drive CE. These broad sheet-like protrusions exert traction on adjacent mesodermal cells causing mediolateral intercalation [257–259]. PCP signaling causes the regulation of cytoskeletal organization that redistributes subcellular PCP components asymmetrically causing polarization of these cells [260]. Moreover, components of the signaling cascade converge or are expressed asymmetrically in the lamellipodia [250, 253].

Among the genes implicated in this net movement of cells, known as convergent extension, are 2 asymmetric molecular systems that control PCP behaviour, the “core” genes and the “Fat-Dachsous” PCP system [261, 262]. The “core” genes give rise to multipass transmembrane proteins: Frizzled (Fzd-3, -6, and -7), Van Gogh (Vangl-1 and -2), Flamingo (Celsr-1, -2, and -3), and cytosolic components, Dishevelled (Dvl-1, -2, and -3), Diego (Inversin), and Prickle (Pk-1 and -2) [263]. The Fat-Dachsous (Ft-Ds) pathway includes the large protocadherins Ft and Ds, acting as its ligand, and Four-jointed (Fj) as a Golgi resident transmembrane kinase [264]. Downstream of the PCP system are PPE (Planar Polarity Effector) genes:* Inturned (In), Fritz (Frtz),* and* Fuzzy (Fy)* [265, 266].* The Multiple Wing Hairs (mwh)* act downstream of both PCP and PPE [267] with Wnt4, Wnt5a, Wnt7a, and Wnt11 as regulators [263].

*Vangl-2* (formerly known as* Ltap* and* Lpp1*) has been identified as the causative gene in the loop-tail mouse [105, 268, 269]. Mutations in* Celsr-1* cause craniorachischisis in the* Crash* mouse [270]. The Dvl-1/Dvl-2, Dvl-2/Dvl-3, Dvl-2/Vangl-2, and Fzd-3/Fzd-6 double knockout mice also have severe NTD forms, mainly craniorachischisis and exencephaly [269, 271–273]. The Vangl-1 and Vangl-2 compound heterozygote exhibits craniorachischisis [274]. The noncore PCP genes also exhibit severe NTD in their mouse mutants including Protein Tyrosine Kinase 7 (PTK7), Scribbled PCP protein, the gene responsible for the* circle tail* mouse phenotype,* Scrib*, and Dishevelled Binding Antagonist of Beta-Catenin 1 (Dact-1) [252, 270, 274–277]. All of these genes have been implicated in the PCP pathway. Failure of convergent extension results in an open neuraxis (the entire neural tube from midbrain to low spine remains exposed) and a shortened embryo, more commonly described as craniorachischisis.

### 9.2. Neural Fold Elevation and Bending

Dorsoventral patterning in the neural development of vertebrates is controlled by the induction and polarizing properties of the floor plate [278]. Expression of various genes such as* sonic hedgehog (Shh), bone morphogenetic protein (BMP) 7, HNF3β*, and* Vangl-1* emanating from the notochord and floor plate is thought to cause cell specification which influences the morphogenesis of the neural tube [106, 107, 112, 113, 252]. The floor plate and notochord appear to control the pattern of cell types that appear along the dorsoventral axis of the neural tube [226, 278]. Morphogenesis of the spinal neural tube, in particular, the formation of the median hinge point (MHP), is most likely a nonneuroepithelial cell autonomous action as it is dependent on the differentiation of ventral cell types by signals transmitted from axial mesodermal cells of the notochord to overlying neuroepithelial cells [278–284].

Implantation and ablation experiments which manipulated the notochord in both chick and mouse embryos [221, 284–287] verified that the notochord is required for formation of the MHP. It was proposed that the notochord releases a morphogen that may regulate MHP formation. Shh protein is expressed in the notochord at this stage [113, 288] and application of either Shh-expressing cells or purified protein to intermediate neural plate explants leads to induction of the floor plate [113], suggesting that Shh is the MHP-inducing morphogen. However, MHP formation is not totally abolished in Shh-null mouse embryos, suggesting that other factors from the notochord may also have MHP-inducing properties [287].

The second site of neural fold bending as described in Section 8.2 and Figure 3(c) is the dorsolateral hinge point (DLHP). Bending of the neuroepithelium at the DLHP is regulated by mutually antagonistic signals external to the neural fold, as reviewed by Greene and Copp [224, 289]. In contrast to midline bending,* Shh* has been shown to inhibit dorsolateral bending in the mouse [287] consistent with an absence of NTDs in Shh-null embryos. Signal(s) arising from the surface ectoderm (SE) comprise(s) a second antagonistic signal involved in the regulation and formation of the DLHPs [290]. This has been suggested as further evidence that bending of the neural folds involves signaling from the SE. Bone morphogenetic proteins (BMPs) are candidates to mediate this signaling. Three BMPs (*BMP2, BMP4*, and* BMP7*) are expressed in the spinal neural tube.* BMP2* and* BMP7* are expressed in the surface ectoderm adjacent to the open spinal neural tube, while* BMP4* is expressed in the surface ectoderm overlying the closed spinal neural tube [291].

Recent studies suggest that* Noggin* may also play a role in regulating DLHP formation [292, 293].* Noggin* is an inhibitor of BMP signaling and is expressed at the tips of the apposing neural folds [293, 294]. Homozygous mouse embryos null for Noggin exhibit both exencephaly and spina bifida (100%) [292, 295]. However, spina bifida does not arise in homozygous Noggin mutants until embryonic day 11-12 when the neural tube ruptures. The spinal neural tube of homozygous null Noggin embryos during neurulation takes on the appearance of a wavy neural tube before the neural tube reopens [293], possibly suggesting an unstable initial closure mechanism.* Shh* works in an antagonistic manner towards* Noggin*, as does* Noggin* towards* BMP* signaling [296]. This suggests that* Noggin* may facilitate bending of the spinal neural tube [293] by overcoming the inhibitory influence of* BMPs*.

Stottmann et al. [293] suggest that the spinal defect in Noggin null embryos results from a failure to maintain a closed neural tube due to a defective paraxial mesoderm [293]. Yip et al. [297] also had shown that the mesodermal extracellular matrix plays an important role in maintaining neuroepithelial rigidity of the spinal neural tube during neurulation [297]. Embryos were cultured in the presence of chlorate, which functions to inhibit sulfation of heparan sulphate proteoglycans (HSPGs) in the extracellular matrix of the mesoderm. This treatment not only resulted in an expedited bending of the DLHPs but also elicited an unnatural shape of neural tube due to a convex shaped mesoderm. However, removal of the paraxial mesoderm does not prevent closure of the spinal neural tube [287].

Interestingly, there are 3 genes which, when mutated, not only affect paraxial mesoderm production in the mouse [109, 292, 298] but also result in an NTD phenotype in the mouse. These are* Cyp26, Noggin*, and* Fgfr1* [94, 109, 293]. The Wnt3a [299], Lef1/Tcf1 double null [108] and Raldh2 [300] mutants also have defective paraxial mesoderm production, with an abnormal neural tube during neurulation. Whether or not the paraxial mesoderm plays a primary role in successful neurulation in these mutants remains unknown.

Neural tube closure does not depend exclusively on the MHP or DLHPs, since closure can occur in the absence of either, as in Mode 3 and Mode 1 spinal neurulation, respectively. However, cell shape changes of some type, affecting morphogenesis of the spinal neural tube, are clearly required for closure to occur in all species studied, including the mouse [254]. Table 5 demonstrates the lack of specific expression of genes at the DLHPs. However, overlapping gene expression throughout the neuroepithelium and tips of neural folds may facilitate the bending mechanism seen in the DLHPs.

### 9.3. Adhesion of the Neural Folds

In all animal species studied, a zone of altered cell morphology with numerous rounded cell blebs has been observed along the tips of the spinal neural folds, immediately prior to adhesion. The observed surface alterations may reflect a change in the properties of the cells at the adhesion site which correlate with initial adhesion between the folds [234, 236, 301, 302]. Structural observations of the point of adhesion in human embryos have yet to be reported, possibly due to insufficient or poor preservation of material so that surface structures cannot be observed.

Adhesion is the final process in the sequence of primary neurulation events. Such physical zippering state of the neural tube has been suggested, in previous studies, as evidence that neural tube closure is a continuous process [303]. However, a debate exists as to whether the physical process of neurulation actually equates to continuous zippering or, more accurately, to a button-like process in which neural tube adhesion initially occurs at various slightly separated points along the axis. According to the latter idea, neural tube adhesion is actually a discontinuous process of closure [222].

PCP regulation may play a role in adhesion and fusion as suggested in both zebrafish and* Xenopus* studies. Firstly, cell division regulated by PCP signaling leads to rescue of neural tube morphogenesis in the trilobite zebrafish mutant [304]. Secondly, the* Xenopus* adhesion molecules, NF-protocadherin, and its cytosolic partner TAF1/Set have been suggested to participate in CE after the neural folds are formed. Disruptions in NF-protocadherin and TAF1 can lead to a shortened AP axis that was not evident until stages 22–25, some time after neural tube closure [305].

Ultrastructures that emanate from the neural folds at the site of closure have been regarded as a secondary process in the frog. This is because wound healing which acts via actin purse-string contraction is thought to be the primary cause of closure in the frog neural tube [306]. Adhesion of the neural tube and epidermis have been suggested to be separate events based upon the observation that the epidermal ectoderm is still able to migrate and cover the open neural tube in both the chick and the frog [302, 305]. However, the issue of whether or not the neural folds could adhere even in the absence of epidermal fusion in both the chick and the frog has yet to be answered.

Adhesion in the neural tube of rodents has been described previously but the mechanism of this highly specialized process is poorly understood [103, 240, 243, 301, 307, 308]. In a recent study, a direct requirement was shown for the binding of a specific ligand (ephrinA5) to a specific type of receptor (EphA7) in order to enable adhesion to occur in the neural tube [243].

Cell to cell adhesion provides impetus for positional cell migration [309]. This may suggest that PCP driven events in the surface ectoderm may play a role in neural tube closure, as suggested in the chick embryo [310]. Epidermal constriction has also been shown to be crucial for spinal neural tube closure in the frog, while the surface ectoderm was shown to be necessary for spinal neural tube closure in the mouse [287, 311].

## 10. Mouse Mutant Models with a Spinal Defect, Not a Neural Tube Defect

Table 4 summarizes the ten mouse mutant models that exhibit a spinal defect alone. Spinal defects encompass mouse mutants with spina bifida (without any other NTD phenotype, e.g., exencephaly and/or craniorachischisis) and abnormal spinal neural tubes with no spina bifida.

The mutants which display only spina bifida are the FGFR1*α* chimeric mutant [94], Traf4 mutant [95], the Shp2 chimeric mutant [96], the axial defects mutant [97], glial cell missing-1 [98], and vacuolated lens [99].

All of these mutants have spina bifida, which denotes incomplete closure of the spinal neural tube. A large majority (4 out 6 of these mutants which have only spina bifida) have a second phenotype that is a second neural tube. Vacuolated lens mutant embryos develop spina bifida and, in addition, an ectopic neural tube is observed, ventral to the open neural tube [99]. In Shp2, FGFR1*α*, and vacuolated lens mutants, an ectopic neural tube is observed during the period of neurulation between E8.5 and E9.5 [94, 96]. In contrast, an ectopic neural tube has only been observed at E12.5 and later stages in Gcm1 mutant embryos [98].

The prevalence of an ectopic neural tube in 2 out of 6 mutants at E9.5–E10.5 seems to suggest that a second neural tube may be a common occurrence and that this predisposition may be the result of an underlying fault in primary neurulation instead of failure of secondary neurulation.

There are many different examples of mouse mutants in which the caudal neural tube is abnormal but the phenotype differs from spina bifida. In many cases, these are described as spinal neural tube defects [100–103]. Apart from the 3 mutants with only spina bifida (Fgfr1, Shp2, and Gcm1) which have 2 neural tubes with one notochord, 2 other mutants with spinal defect but no spina bifida share the same predicament. These are the EphA2 null mouse [101] and PAK4 null mouse [100]. Another abnormal spinal neural tube phenotype is a wavy spinal neural tube that occurs in the WASP null mouse and the Vinculin null mouse [102, 103]. Vinculin is a large protein that binds multiple cytoskeletal proteins, actin, *α*-actinin, talin, paxillin, VASP, ponsin, vinexin, and protein kinase C (PKC) which have been suggested to be the adhesion scaffold that connects early adhesion sites to actin-driven protrusive machinery in enabling motility [312].

Abnormal and ectopic spinal neural tubes may be regarded as variant forms of NTDs as it may be possible that the neural tube reopens after closure due to various reasons. Ectopic neural tube may take on many different variations apart from the expected second or multiple neural tubes. Among them are a neural tube positioned above another neural tube as well as a wavy neural tube phenotype that is observed in many knockout mice with NTDs. The wavy region in these knockout mice has not had its spinal neural tube sectioned; thus it remains unknown whether the neural tube remains adhered. Spina bifida occulta in humans is usually accompanied by various physical abnormalities such as lipoma, rachischisis, hair tufts, ectodermal sinuses, skin pigmentation, or diastematomyelia. These associated defects occur in either syndromic or nonsyndromic NTDs. However, they may be missed and not categorized properly in cases of transgenic mice with possible NTDs. There is only one example of a null mouse in which these abnormalities have been well described which is the Gcm1 mouse mutant that exhibits both open (meningomyelocele) and close (lipoma and diastematomyelia) spina bifida in its litters [98].

## 11. Haploinsufficiency in Mouse and Man

Haploinsufficiency is poorly studied in both man and mouse. Furthermore, the study of the occurrence of spina bifida in genes acting in an additive or subtractive manner is almost unknown. Currently, there are 5 studies in the mouse, which have demonstrated spina bifida and the interaction of the involved genes mechanistically. These include* Lrp6* and* Wnt5a* [313],* Zac1* and* Suz12* [314],* Hira* and* Pax3* [315],* Rybp* encompassing* Ring1* and* YYP1* [316], and haploinsufficiency of the components in the primary cilium of the hedgehog pathway [317].

The scenario in humans is somewhat similar in that there are 4 studies to date demonstrating the involvement of haploinsufficiency in the causation of spina bifida. The* Pax3* gene and the* EphA4* gene act in concert with each other in causing spina bifida due to interstitial deletion at position 2q36 [318]. Furthermore, in the same paper, Goumy et al. [318] suggested that a similar phenomenon occurs in the mouse when taking into account the spina bifida phenotype seen on the Splotch mouse that is affected by both* Pax3* [93] and* EphA4* [319], albeit the link between the two in the mouse has yet to be ascertained. The hedgehog pathway has also been implicated in humans, where spina bifida occurs when Patched is perturbed when implicated with Gorlin syndrome [320]. The third and fourth studies implicating human spina bifida involve haploinsufficiency in the region of 13q [321] and 7q [322].

## 12. Conclusion

This review paper aims to probe spina bifida, the surviving form of neural tube defects, closely and to analyze the relationship of what can be learnt from the mouse model of spina bifida and to use that knowledge in order to shine a brighter understanding with regard to the human form.

What is very obvious is that there have been a multitude of genes (74 according to this review) which regulate specifically spina bifida in the mouse. This is a very high number of genes; therefore the take home message would be in our opinion that there are a multitude of genes that can, if perturbed, cause spina bifida. Whether or not these genes cause the condition or are in fact a player in a pool of numerous genes, which can do the job of closing the spinal neural tube, is a tantalising idea. Therefore, we put forth the idea that perhaps these 74 may be working with other genes in their family or other genes which share a common pathway in order to close the neural tube. Furthermore, the idea of gene-gene interaction which promotes heterogeneity among genes is incomplete without also considering the idea of haploinsufficiency of genes, where many mutations in mankind are somehow protected from having a deleterious phenotype by having other genes compensate the job of the gene or genes being perturbed. A very good example of this would be the Vangl-1 and Vangl-2 compound heterozygote mouse mutant which lacks a single allele of both Vangl-1 and Vangl-2; therefore the probability that the 2 genes compensate each other is high and both genes are required in a certain amount of dose, lack of which translates into a neural tube defect phenotype. Therefore, the mouse model which examines the delineation of genes has not completed its true worth until scientists understand the biology of the disease or condition better by also taking into account (i) the amount (the functioning allele) of the said gene and (ii) the interaction with other genes in its family which may be able to compensate its function as well as (iii) the interaction with other genes which share a common pathway. The mouse is a powerful tool to study spina bifida because it is a mammal like humans and its embryology is similar to humans and therefore it is an indispensable tool to mechanistically study the structural changes involved in spinal neural tube closure. The genes involved in spinal neural tube defects may differ in man and mouse; however, parallels may be drawn between the principles of how the genes interact in influencing spinal neural tube closure in both man and mouse.

## Figures and Tables

**Table 1:** Comprehensive list of syndromic spina bifida.

Mode of inheritance	Condition	References
Autosomal recessive	(1) Jarcho-Levin syndrome (spondylocostal dysostosis): shortened trunk, opisthotonus position of the head, short neck, barrel-shaped thorax, multiple wedge shaped and block vertebrae, spina bifida, and rib anomalies. (2) Cerebrocostomandibular syndrome: Pierre Robin anomaly, speech difficulties, severe micrognathia with glossoptosis, small thorax with rib-gap defects, occasional intellectual impairment, and spina bifida. (3) Human athymic nude/SCID: T-cell defect, congenital alopecia, nail dystrophy, and spina bifida. (4) Neu-Laxova syndrome: spina bifida, severe intrauterine growth retardation, microcephaly, protruding eyes, abnormal skin, and limb defects. (5) PHAVER syndrome: spina bifida, pterygia, heart defects, segmentation defects of the spine, and radioulnar synostosis.	[23–35]

Autosomal dominant	(1) DiGeorge syndrome: hypocalcemia, parathyroid hypoplasia, thymic hypoplasia, conotruncal cardiac defects, and facial features. A case of associated spina bifida was reported. (2) Waardenburg syndrome: Type I, wide bridge of the nose, lateral displacement of the inner canthus, pigmentary disturbance of frontal white blaze of hair, heterochromia iridis, white eye lashes, leukoderma, cochlear deafness, and spina bifida. Type III, partial albinism, blue eyes, deaf-mutism, undeveloped muscles, fused joints in the arms, skeletal dysplasia, and spina bifida. (3) Sacral defect with anterior meningocele (SDAM): sacral agenesis and spina bifida. (4) Czeizel-Losonci syndrome: split hand/split foot, hydronephrosis, and spina bifida.	[36–45]

X-Linked	(1) Focal dermal hypoplasia (male lethality, atrophy and linear pigmentation of the skin, papillomas of skin and mucosae, ocular defects, hypoplastic teeth, and digital anomalies apart from spina bifida). (2) Zic3 (spina bifida with abdominal situs inversus, complex cardiac defects, asplenia, and polysplenia). (3) Congenital hemidysplasia with ichthyosiform erythroderma and limb defects (CHILD syndrome).	[46–48]

Sporadic	(1) Isolated hemihyperplasia: asymmetric overgrowth of one or more regions with one reported case of lumbar myelomeningocele. (2) Diprosopus: conjoined twins consisting of one neck, one body, and a single hand with various forms of duplication of the craniofacial structures. May be associated with spina bifida. (3) Pentalogy of Cantrell: midline supraumbilical abdominal wall defect, defect of the lower sternum, defect of the diaphragmatic pericardium, deficiency of the anterior diaphragm, and congenital cardiac anomalies. Spina bifida has been reported. (4) Weissenbacher-Zweymüller syndrome: congenital neonatal rhizomelic dwarfism, metaphyseal widening of the long bones, vertebral coronal clefts, micrognathia, cleft palate, depressed nasal root, hypertelorism, protruding eyes, occasional sensorineural deafness, and spina bifida.	[49–53]

**Table 2:** List of mouse models that exhibits spina bifida (reviewed in [4, 54]).

Mouse models	
Gene or mutants (53 genes)	
(1) *Aln*	
(2) *Ambra1*	
(3) *Apapb*	
(4) *Axd *mutant	
(5) *Axin1*	
(6) *Cyp26a1*	
(7) *Ds* mutant	
(8) *Dvl2*	
(9) *Fgfr1 *chimera	
(10) *Fkbp8* hypomorph	
(11) F*pn1*	
(12) *F2r*, *F2rl1* (*Par1*, *Par2*) (digenic)	
(13) *Grhl3*	
(14) *Gnaz*^−/−^; *Grhl3*^*Cre/+*^ (digenic)	
(15) ^*g*^*Gpr161* (*vl* mutant)	
(16) *ct* mutant (*Grhl3 *hypomorph)	
(17) *Itgb1*	
(18) *ltpk1*	
(19) *Lrp6*	
(20) *Lrp6* (rs mutant, hypomorph)	
(21) *Map3k4*	
(22) *Mapk4* partial function	
(23) *Marcksl1 (Mlp)*	
(24) *Med12*	
(25) *Msgn1*	
(26) *Msx1, Msx2* (digenic)	
(27) *Ndst1*	
(28) p*28IP*	
(29) *Pax3* (*Sp* mutant)	
(30) *Pax3 (Sp*^*2H*^)	
(31) *Pax3 (Sp*^*d*^)	
(32) *Ptpn9 (Meg2)*	
(33) *Rab23* (*opb* mutant)	
(34) *Rab23 (opb*^*2*^)	
(35) *Rac1*^−/−^; *Grhl*3^*Cre*/+^ (digenic)	
(36) *Sfrp1*, *Sfrp2* (digenic)	
(37) *Shroom3*	
(38) *Sp8*	
(39) *Spint2* (HA12)	
(40) *T *(*T*^*c*^*/t*^*w5*^ mutant)	
(41) *Terc*	
(42) *Traf4*	
(43) *Trpm6*	
(44) *Tulp3*	
(45) *Tulp3* (*hhkr* mutant)	
(46) *vl* mutant	
(47) *Wnt3a* (*vt* mutant hypomorph)	
(48) *Zfhx1a*	
(49) *Zic2* hypomorph	
(50) *Zic2*^*Ku*^	
(51) *g2e*	
(52) *1B*	
(53) *97c2*	
Mouse strain (1 gene)	
(54) *NOD*	
PCP genes (4 genes)	
(55) *Lp*, *Crc* mutants (digenic, heterozygous)	
(56) *Lp*, *Ptk7 *(digenic, heterozygous)	
(57) *Vangl*^*Lp/*+^, *Sec24b*^+/−^ (digenic)	
(58) *Vangl*^*Lp/+*^, *Sfrp1*^−/−^, *Sfrp2*^+/−^, *Sfrp5*^−/−^ (4 genes)	
Lethal before gestation day 12 (3 genes)	
(59) *Brca1*	
(60) *Fgfr1 *(alpha isoform)	
(61) 22C	
Spina bifida occulta (13 genes)	
(62) *Foxc1* (*ch* mutant)	
(63) *Foxc2*	
(64) *Lrp6*	
(65) *Nog*	
(66) *Pdgfra*	
(67) *Pdfgc*	
(68) *Pkd1*	
(69) *Prrx1*	
(70) *sno* mutant	
(71) *Tgfb2*	
(72) *T *(*T*^*c/+*^ mutant)	
(73) *Traf4*	
(74) *Zic1*	
Mouse models studied in nutrient supplement rescue (5 genes)	
(1) *Sp*^*2H*^mutant at *Pax3 *gene	
(2) *Sp* mutant at *Pax3 *gene	
(3) *Axd* mutant	
(4) *ct* mutant (hypomorph at *Grhl3*)	
(5) *Grhl3* null	

**Table 3:** Comprehensive list of human spina bifida genes.

Genes (40 genes)	Population and sample size (in brackets) of spina bifida cases		References
	Studies showed association with genes related NTDs or risk factor for spina bifida	Studies showed no association with genes related NTDs	
One carbon metabolism (including homocysteine remethylation) (8 genes)			
*ALDH1L1*	Dutch (180 patients)		[55]
*BHMT*	Mixed USA (259 cases)	Mixed USA (252 cases), Dutch (180 patients)	[55–57]
*CHKA*	Mixed USA (103 cases)		[58]
*MTRR*	Mixed USA (259 cases), mixed UK (229 patients), Dutch (109 cases)	Irish (575 mixed families), Dutch (109 cases; 180 patients)	[55, 56, 59–61]
*NOS3*	Mixed USA (301 families), Dutch (109 cases)	Mixed USA (259 cases), Dutch (180 patients)	[55, 56, 62, 63]
*PYCT1A*	Mixed USA (103 cases)		[58]
*SARDH*	Dutch (180 patients)		[55]
*TRDMT1*	Dutch (180 cases)		[55]

NTDs in mouse mutant (7 genes)			
*BRCA1*	Mixed USA (268 patients and parents)		[64]
*CFL1*	Mixed USA (246 cases)		[65]
*PAX3*	USA (74 cases)		[66]
*PDGFRA*	Dutch (88 cases and 56 mothers)	Mixed USA (407 triads)	[67, 68]
*TXN2*	Mixed USA (48 cases)		[69]
*ZIC2*	Dutch (117 mixed patients)		[70]
*ZIC3*	Dutch (117 mixed patients)		[70]

Folate metabolism (5 genes)			
*CBS*	Mixed USA (259 cases)	Dutch (180 patients)	[55, 56]
*DHFR*	Irish (283 cases), mixed USA (61 cases, multiaffected families)	Mixed USA (259 cases), mixed UK (229 patients), Dutch (180 patients; 109 patients)	[55, 56, 71–73]
*MTHFD1*	Irish (509 mixed cases), mixed USA (259 mixed cases), Italian (142 cases), Irish (176 mixed cases)	Mixed UK (229 patients), Dutch (103 cases), Dutch (180 patients)	[55, 56, 59, 62, 71, 74–76]
*MTHFR*	Irish (451 cases), mixed USA (259 cases), mixed UK (229 patients), Italian (15 cases)	Dutch (180 patients), Mexican (Yucatan) (97 cases), Italian (15 cases)	[55, 56, 59, 60, 77, 78]
*TYMS*	Non-Hispanic white USA (264 cases), mixed USA (259 cases)	Dutch (180 patients)	[55, 56, 79]

Glucose metabolism (4 genes)			
*GLUT1*	Mixed USA (507 cases)		[80]
*HK1*	Mixed USA (507 cases)		[80]
*LEP*	Mixed USA (507 cases)		[80]
*LEPR*	Mixed USA (507 cases)		[80]

DNA repair and DNA methylation (3 genes)			
*APE1*	Mixed USA (380 patients)		[81]
*XPD*	Mixed USA (380 patients)		[81]
*SOX18*	Belgium (83 patients)		(Rochtus et al., 2016)

Folate transport (2 genes)			
*CUBN*	Dutch (179 patients)		[55]
*SLCA19A1, RFC-1*	Dutch (180 patients)	Mixed USA (259 cases), mixed UK (229 patients)	[55, 56, 59]

PCP genes (4 gene)			
*VANGL1*	Italian and mixed USA (658 patients), Italian and French (102 patients)	Mixed UK and USA (24 patients)	[44, 82, 83]
*CELSR1*	California (192 patients)		[84]
*SCRIB*	California (192 patients)		[85]
*DVL1*	Han Chinese cohort (20 cases)		[86]

Retinol metabolism (1 gene)			
*ALDH1A2*	Mixed USA (318 families)		[87]

Axial development in mouse (1 gene)			
*T (brachyury)*	Mixed USA (316 cases)		[88]

Methylation reactions (1 gene)			
*PCMT1*	Mixed USA (152 cases)	Dutch (180 patients)	[55, 89]

Oxidative stress (2 genes)			
*SOD1*	Mixed USA (610 trios or duos)		[90]
*SOD2*	Mixed USA (610 trios or duos)		[90]

Intermediate filament protein (1 gene)			
*LMNB1*	Mixed UK, USA, and Swedish (233 patients)		[91]

Cell adhesion molecules (1 gene)			
*NCAM1*	USA (204 patients)		[92]

A total of 40 genes reported showing association/risk factor for spina bifida as reviewed in Greene et al. [93].

**Table 4:** 

Mutant name	Gene mutated	Function of protein	Possible mechanism of NTD	Schematic representations of ectopic spinal neural tube	Rate of occurrence of spina bifida	Phenotype and reference
Fibroblast growth factor receptor 1 (knockout producing chimeras)	*Fgfr1*	Growth factor receptor	Unknown (NTDs occur only in chimaeras)	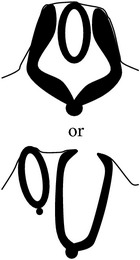	E10.5, 29.5% have spina bifida and 15% have ectopic neural tube	Spina bifida, second NT; NT in NT & kinky tail [94]

Tumour necrosis factor receptor associated factor 4 (knockout)	*Traf4*	Intracellular signaling adaptor	Unknown	No ectopic neural tube	40% homozygous nulls have spina bifida	Spina bifida [95]

Shp2 (knockout producing chimeras)	*Shp2*	Tyrosine phosphatase (dephosphorylates proteins)	Unknown (NTDs occur only in chimaeras)	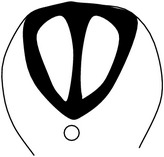	E10.5, 36% of high content chimeras have second neural tube and 59% have spina bifida	Spina bifida, second NT [96]

Axial defects (spontaneous mutant; gene not identified)	ND	ND	ND	No ectopic neural tube	10% penetrance in CD1	Spina bifida [97]

Glial cells missing-1 (knockout)	*Gcm1*	Transcription factor	Ectopic expression causes NTDs by unknown mechanism	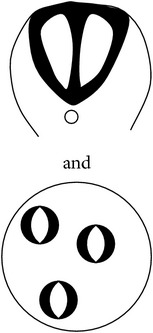	25.8% transgenics have spina bifida; 100% transgenics have ectopic neural tube	Spina bifida; multiple NT [98]

Vacuolated lens (spontaneous mutant)	ND	ND	Suggested failure in apposition and fusion?	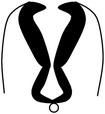	50% of homozygous nulls show spina bifida at 12 dpc	Spina bifida [99]

PAK4 (knockout)	*PAK4*	Cytoskeletal organization	Target for Rho GTPase Cdc42	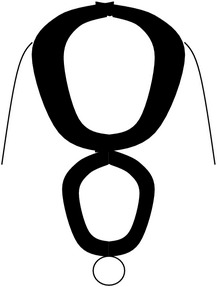	None	Double neural tube with one notochord No spina bifida [100]

EphA2 (knockout)	*EphA2*	Adhesion and fusion?	Receptor tyrosine kinase	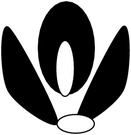	None	Kinky tail with double neural tube No spina bifida [101]

WASP (knockout)	*WASP*	Cytoskeletal organization	Formation of cell-surface projections (filopodia) required for cell movement and actin-based motility	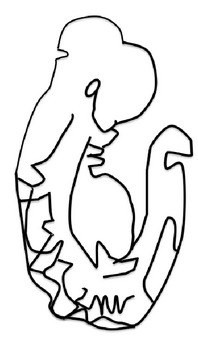	None	Wavy neural tube No spina bifida [102]

Vinculin (knockout) (E10 Lethal)	*Vinculin*	Cytoskeletal organization	Major constituent of cell junctions (cell matrix & cell-cell)	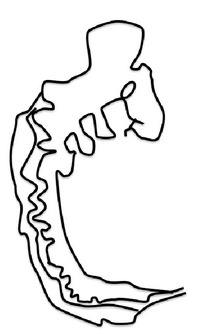	None	Wavy neural tube No spina bifida [103]

**Table 5:** 

Neural tube structure	Genes expressed at 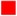
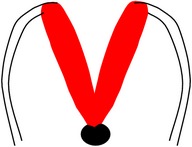	Neuroepithelium *Zic2 *[104] *Vangl2 *[105]
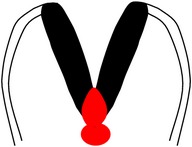	Floor plate and notochord *HNF3 *[106] *Vangl1 *[107] *Wnt3a* [108] Cyp26a1 [8, 109] *Shh *[11] Map3k4 [110] Marcksl1 (Mlp) [18, 111] Traf4 [21, 95]
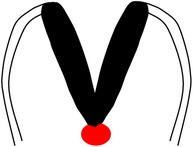	Notochord *BMP7* [112] *Brachyury* [6] *Shh *[10, 11, 113, 114]
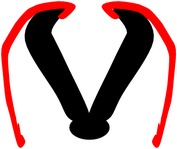	Surface ectoderm *Fgf8 *[115] *Grainyhead-like 2* [116] *BMP7* [104] *Wnt6* [104] *Notch1* [108]
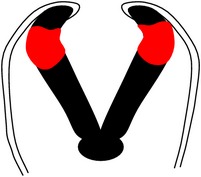	Dorsolateral hinge points None
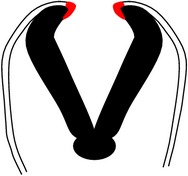	Tips of neural folds at E9.5 *Axin2* [117] *Pax3* [118]
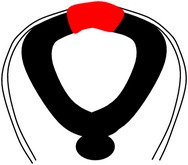	Dorsal roof of closed neural tube bridge *Zic2* [104] *Msx1* [104] *Wnt1* [9, 119] *BMP6* [119]
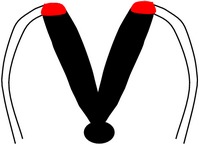	Tips of neural folds (surface ectoderm) *Grainyhead-like 3 *(in neural ectoderm at E8.5) [116] *Par1 *and *Par2* [120]
